# Annexin-A1 peptide down-regulates the leukocyte recruitment and up-regulates interleukin-10 release into lung after intestinal ischemia-reperfusion in mice

**DOI:** 10.1186/1476-9255-10-10

**Published:** 2013-03-13

**Authors:** Bruna Candido Guido, Marianna Zanatelli, Wothan Tavares-de-Lima, Sonia Maria Oliani, Amílcar Sabino Damazo

**Affiliations:** 1 Department of Biology; Institute of Biosciences, Letras e Ciências Exatas (IBILCE), São Paulo State University (UNESP), São José do Rio Preto, SP, 15054-000, Brazil; 2 Department of Pharmacology, Institute of Biomedical Sciences (ICB), University of São Paulo (USP), São Paulo, 05508-900, Brazil; 3 Department of Basic Science in Health; Faculty of Medicine (FM), Federal University of Mato Grosso (UFMT), Mato Grosso, MT, 78060-900, Brazil

**Keywords:** Annexin-A1, Lung, Macrophage, Neutrophil, Interleukin-10 (IL-10)

## Abstract

**Background:**

Intestinal ischemia/reperfusion (IR) injury is a serious and triggering event in the development of remote organ dysfunction, from which the lung is the main target. This condition is characterized by intense neutrophil recruitment, increased microvascular permeability. Intestinal IR is also responsible for induction of adult respiratory distress syndrome, the most serious and life-threatening form of acute lung injury. The purpose of this study was to investigate the effect of annexin-A1 protein as an endogenous regulator of the organ remote injury induced by intestinal ischemia/reperfusion. Male C57bl/6 mice were subjected to intestinal ischemia, induced by 45 min occlusion of the superior mesenteric artery, followed by reperfusion.

**Results:**

The intestinal ischemia/reperfusion evoked a high intensity lung inflammation as indicated by the number of neutrophils as compared to control group. Treatment with annexin-A1 peptidomimetic Ac2-26, reduced the number of neutrophils in the lung tissue and increased its number in the blood vessels, which suggests a regulatory effect of the peptide Ac2-26 in the neutrophil migration. Moreover, the peptide Ac2-26 treatment was associated with higher levels of plasma IL-10.

**Conclusion:**

Our data suggest that the annexin-A1 peptidomimetic Ac2-26 treatment has a regulatory and protective effect in the intestinal ischemia/reperfusion by attenuation of the leukocyte migration to the lung and induction of the anti-inflammatory cytokine IL-10 release into the plasma. The anti-inflammatory action of annexin-A1 and its peptidomimetic described here may serve as a basis for future therapeutic approach in mitigating inflammatory processes due to intestinal ischemia/reperfusion.

## Background

Intestinal ischemia/reperfusion (I/R) injury is a serious and triggering event in the development of remote organ dysfunction, of which the lung is the main target. Indeed, intestinal I/R is a well recognized event involved in acute lung injury (ALI) induction [1]. This condition is characterized by intense neutrophil recruitment, increased microvascular permeability and is responsible for induction of adult respiratory distress syndrome (ARDS), the most serious and life-threatening form of acute lung injury [2].

Local injury associated with intestinal I/R are responsible for the release into the blood stream of IL-1β, TNF-α, prostanoids, oxygen and nitrogen-derived free radicals. All of these mediators play pivotal role in the systemic inflammation [3]. Experimental and clinical evidences suggest that systemic inflammation contributes to the induction of pulmonary microvascular permeability, neutrophil influx and lung function deterioration [4,5].

Moreover, neutrophil migration from blood into lung compartment is a significant event for acute lung injury induction [6]. However, endogenous mechanisms underlying neutrophil trafficking after intestinal I/R are still unclear. Thus, it is important to employ new approaches in order to understand activation and sequestration of neutrophils in the intestinal ischemic events [7].

The anti-inflammatory protein annexin-A1 (ANXA1) is a potent mediator of inflammation resolution and a 37-kDa member of the annexin family of calcium and phospholipid-binding proteins, expressed constitutively in many cells, including neutrophil gelatinase granules [8]. Exogenous AnxA1 or its N-terminal peptidomimetic (Ac2-26) administration has been shown to elicit protective anti-inflammatory actions via both *in vitro* and *in vivo* anti-neutrophil migration mechanisms [8-12]. Moreover, AnxA1 has been shown to have cardio protective effects against myocardial ischemia and reperfusion injury in rats and mice, at least in part due to its inhibitory actions on neutrophils [10,13]. However, the intracellular mechanisms involved in these actions have not been fully elucidated [14]. Because of the difficulties in producing a biologically active recombinant ANXA1 protein, its N-terminal peptide has been commonly used for *in vivo* and *in vitro* studies. The peptide Ac2-26 shares many of the anti-inflammatory activities of ANXA1 and as the name suggests, comprises the acetylated N-terminal sequence of ANXA1 [12,13]. Thus, we hypothesize that organ remote injury induced by intestinal I/R (notably acute lung injury) could be influenced by endogenous control by AnxA1.

## Material and methods

### Animals

Male C57bl/6 mice (20–25 g of body weight), maintained on a standard chow pellet diet with tap water *ad libitum* were used for all experiments. Animals were housed at a density of five animals per cage in a room with controlled lighting (lights on from 8:00 a.m. to 8:00 p.m.) and temperature (21–23°C). Experiments were performed according to Committee of Ethics in Animal Research, FAMERP, SP, Brazil (CEEA; Protocol 585107) and in conformity with the directives of the European Union.

### Intestinal ischemia/reperfusion model

Laparotomy was carried out in mice (n = 10) under anesthesia with ketamine® [dopalen, Vetbrands, Brazil, 18.6 mg/Kg, intramuscular (i.m.)] and xylasine® (anasedan, Vetbrands, Brazil, 2.3 mg/Kg, i.m.). The superior mesenteric artery was exposed through a midline abdominal incision and occluded using a microsurgical clip [15]. After 45 min of arterial occlusion, the clip was removed and intestinal perfusion was re-established. The animals were sacrificed 2 h and 24 h later by exsanguination, via the abdominal aorta, under deep anesthesia. The sham operated group (n = 10) consisted of mice submitted to the same surgical procedures including mesenteric artery dissection but not submitted to the arterial occlusion. An additional group of non-manipulated mice (n = 10) was added to obtain normal values of the variables studied.

### Peptide Ac2-26 treatment

Groups of mice (n = 10) were pretreated with the peptide Ac2-26 (Ac-AMVSEFLKQAWFIENEEQEYVQTVK; Invitrogen, USA) 1 mg/kg ip.1 h before the intestinal IR. As a negative control, mice were treated with Ac2-26 (i.p.), or vehicle (PBS) alone.

### Blood and bronchoalveolar cell counts

At 2 h and 24 h post-reperfusion, mice were sacrificed as indicated above and blood samples (100 μL) obtained from the abdominal artery were diluted 1:10 in Turk’s solution (0.1% crystal violet in 3% acetic acid) for cell count using a plastic syringe (1 mL). In a parallel set of experiments, brochoalveolar lavage (BAL) was performed according to Riffo-Vasquez et al. [16]. In brief, after semi-excision of the trachea, a plastic cannula was inserted and the lung was washed with 1 mL of saline solution (0.9%) containing 6 mM sodium citrate. This operation was repeated twice. BAL was centrifuged (600 g for 10 min, 4°C), and the cell-free supernatants were frozen at −80°C for subsequent cytokine analysis. An aliquot of cell-free supernatant was used to analyze the protein concentrations in BAL fluid using Bradford assay kit (Sigma, St. Louis, USA). Cell pellets were re-suspended with PBS, and an aliquot (190 μL) was diluted 20:1 in Turk’s solution (3% crystal violet in 20% acetic acid) for cell counts. Total and differential counting was obtained using a Neubauer chamber utilizing a x40 objective upon light microscope Axioskop II mot plus (Zeiss, GR). BAL cells were distinguished as polymorphonuclear (PMN), monocyte/macrophage and lymphocytes, whereas peripheral blood cells were classified as PMN, peripheral blood mononuclear cells (PBMN) and lymphocytes. All analyses were done by two blinded investigators.

### Pulmonary microvascular leakage

Pulmonary vascular permeability was assessed by Evans blue dye extravasation. In brief, Evans blue dye (25 mg/Kg) was given intravenously to mice (n = 5 per group) 5 min before the animals were killed. Then, the lungs perfused (via the pulmonary artery with pH 7.0 PBS containing 5 IU/mL heparin) and two samples of lung parenchyma removed. Both were weighted and then one was placed in formamide (4 mg/mL wet weight) at 20°C for 24 h and the other was put to dry in oven (60°C) till >constant weight. The concentration of Evans blue dye extracted in formamide was determined by spectrophotometry at a wavelength of 620 nm (Bio-Tek Instruments, USA) using standard dilution of Evans blue in formamide (0.3–100 mg/mL). The dry/wet ratio of each lung sample was determined (index of edema) and used in the final calculation of Evans blue extravasation which was expressed as mg Evans blue/100 g of dry weight. The expression of the results as a function of dry weight of tissue avoided under-evaluation of changes due to edema.

### Lung myeloperoxidase (MPO) activity

MPO was measured as an index of the presence of neutrophils. Lung tissue samples were obtained from mice killed after 2 or 24 h of intestinal reperfusion (n = 5 per group). The lungs were perfused via the pulmonary artery with pH 7.0 phosphate buffered saline (PBS) containing 5 IU/mL heparin. Briefly, to normalize the pulmonary MPO activity among the group, whole lung was homogenized with 3 mL/g PBS containing 0.5% of hexadecyltrimethylammonium bromide and 5 mM EDTA, pH 6.0. The homogenized samples were sonicated (Vibra Cell, Sonics Materials, USA) for 1 minute and were then centrifuged at 37,000 g for 15 min. Samples of lung homogenates (20 mL) were incubated for 15 min with H_2_O_2_ and ortodianisidine; the reaction was stopped by the addition of 1% NaNO_3_. Absorbance was determined at 460 nm using a microplate reader (Bio-Tek Instruments, USA).

### Histopathological analysis

In a new set of experiments, after euthanasia pulmonary artery was perfused (20 mL of PBS) in a retrograde direction in order to remove the intravascular blood from the lung. The lungs were inflated with air, to avoid alveolar collapse, and fixed in 4% paraformaldehyde, 0.5% glutaraldehyde and 0.1 M sodium phosphate buffer (pH 7.4) for 2 h at 4°C. The lungs were then fragmented, washed, dehydrated in ethanol and embedded in LR Gold resin (London Resin, UK). Sections were cut (1 μm thick) (Leica RM2265, Leica, GR), mounted on slides, and stained with toluidine blue. Quantification of leukocytes in tissue samples was performed with a high-power objective (x40) on Zeiss-Axioskop 2 light microscope (Carl Zeiss, Jena, Germany), and measures of the area of analysis was done with the software Axiovision (Zeiss, GR). Data was reported as cells/mm^2^ (analyzing at least 10 distinct sections per mouse). All analyses were done by two blinded investigators.

### Interleukin 10 and tumoral necrosis factor (TNF)-α quantification

Aliquots of blood fluids were centrifuged at 4,000 g for 10 min. Anti-inflammatory cytokine IL-10 and TNF-α concentrations were measured using specific enzyme-linked immunosorbent assay kits purchased from R&D System (Abingdon, UK).

### Annexin-A1 expression by immunohistochemistry and western blotting

Lung immunohistochemistry to detect AnxA1 protein was performed [17]. Sections were blocked with 10% bovine serum albumin in PBS (PBSA) followed by overnight incubation at 4°C with the polyclonal rabbit anti-AnxA1 antibody (1:200 in 1% PBSA; Invitrogen, USA). As a negative control of the reaction, some sections of lungs were incubated with non-immune rabbit serum (1:200 working dilution; Sigma-Aldrich) instead of the primary antibody. A goat anti-rabbit IgG (Fc fragment-specific) antibody conjugated to 5 nm colloidal gold (1:100; British BioCell International, UK) was then used as secondary antibody and incubated for 1 h at room temperature. Silver enhancing solution (British BioCell International) was used to augment gold particle staining and sections were counterstained with haematoxylin. Densitometry was conducted using the Axioskop II microscope (Zeiss, Germany) and Axiovision (Zeiss) software was used to determine protein intensity of the sample (arbitrary 0–255 U scale).

To analyze AnxA1 immunoreactivity in the tissue, lung fragments were homogenized in EDTA free protease inhibitor (Roche, UK). Protein concentration was determined by the Bradford assay [18]. Equal protein amounts (30 μg) were diluted with sample buffer and electrophoresed in a 10% polyacrylamide gel in running buffer (0.3% Tris base, 1.44% glycine, 0.1% SDS in distilled water). Proteins were transferred onto Hybond-C extra nitrocellulose membranes with a transfer buffer (0.3% Tris base, 1.44% glycine, 20% methanol in distilled water). Membrane was initially blocked with 5% non-fat milk solution in TBS containing 0.1% Tween 20, followed by incubation with an antibody anti-AnxA1 to detect both cleaved (33 kDa) and intact protein (37 kDa) (1:1000; Invitrogen, USA). The samples were incubated with HRP-linked anti-mouse secondary antibody (1:2000; Amersham Biosciences, USA) and the signal was amplified with ECL kit (Western blotting detection reagent; Amersham Biosciences, USA) and visualized with a photographic film (Hyperbond, Amersham Biosciences). This experiment was performed five times with the animals of the experimental groups.

### Statistical analysis

In all cases, data are reported as mean ± SEM of five mice per group. Statistical differences between groups were determined by one way ANOVA followed by the Bonferroni test. Values of P <0.05 were considered significant.

## Results

### Effect of Ac-2-26 treatment on the leukocyte kinetics in blood and bronchoalveolar lavage after intestinal I/R

**Figure 1:**
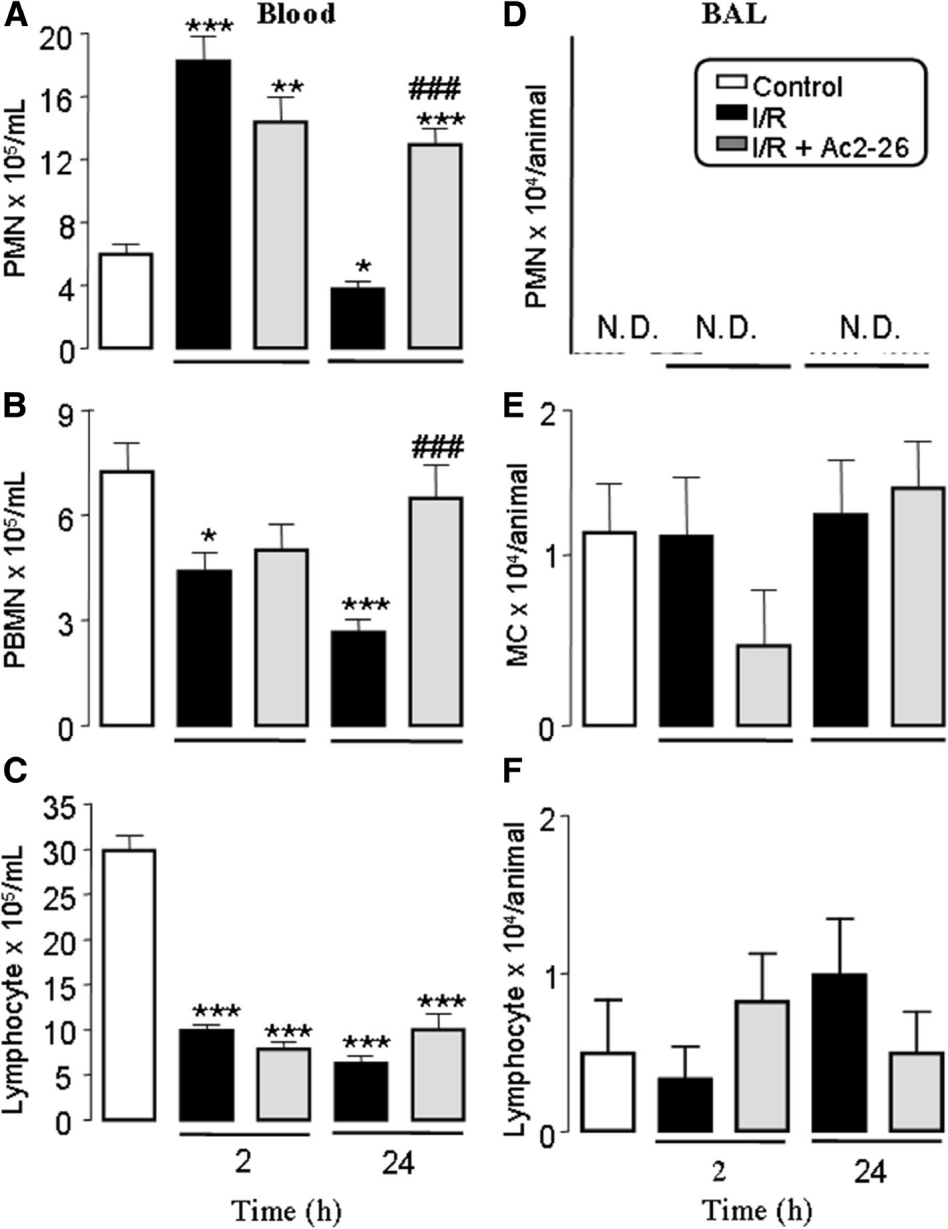
**Time course of leukocyte influx into the blood.** Mice (n = 10 per group) were submitted to intestinal ischemia for 45 min, followed by reperfusion at 2 and 24 h. At different time points, blood aliquots were collected for polymorphonuclear (PMN) (**A**), peripheral blood monocyte (PBMN) (**B**) and lymphocyte (**C**) quantification. BAL was also performed to measure PMN (**D**), monocyte/macrophage (MC) (**E**) and lymphocytes number (**F**). Data are mean ± SEM from two separate experiments with five mice each. *P < 0.05, **P < 0.01 and ***P < 0.001 versus control group values; ###P < 0.01 versus corresponding intestinal I/R group values.

The number of peripheral blood neutrophils was significantly increased 2 h after reperfusion. However, 24 h later, the number of these cells was similar to those in the control group (naive group: 6.1 ± 0.4; sham group: 6.0 ± 0.2; negative control group saline: 6.1 ± 0.2; negative control group Ac2-26: 5.9 ± 0.4 × 10^5^/mL). On the other hand, PBMN and lymphocytes numbers were reduced (2 and 24 h) after reperfusion compared to control group (Figure 1 panels A-C).

Pretreatment with peptide Ac2-26 had no effect on the increases in the values of peripheral blood leukocytes 2 h after reperfusion. In contrast, 24 h after reperfusion, Ac2-26 treatment prevented the reduction of circulating PBMN and PMN numbers as found in non-treated groups of mice upon intestinal I/R (Figure 1A and 1B). On the other hand, circulating lymphocytes number was not affected by pretreatment with peptide Ac2-26 (Figure 1C).

In order to verify the effects of intestinal I/R on the cell recruitment into bronchoalveolar compartment, we quantified the cells in BAL, 2 h and 24 h after reperfusion. As shown in Figure 1 PMN were not detected (Figure 1D), whereas monocyte/macrophages and lymphocytes number were not altered (Figure 1E and 1F).

### Edema and leukocytes infiltration in the lung after intestinal I/R induction and Ac2-26 treatment in mice

**Figure 2:**
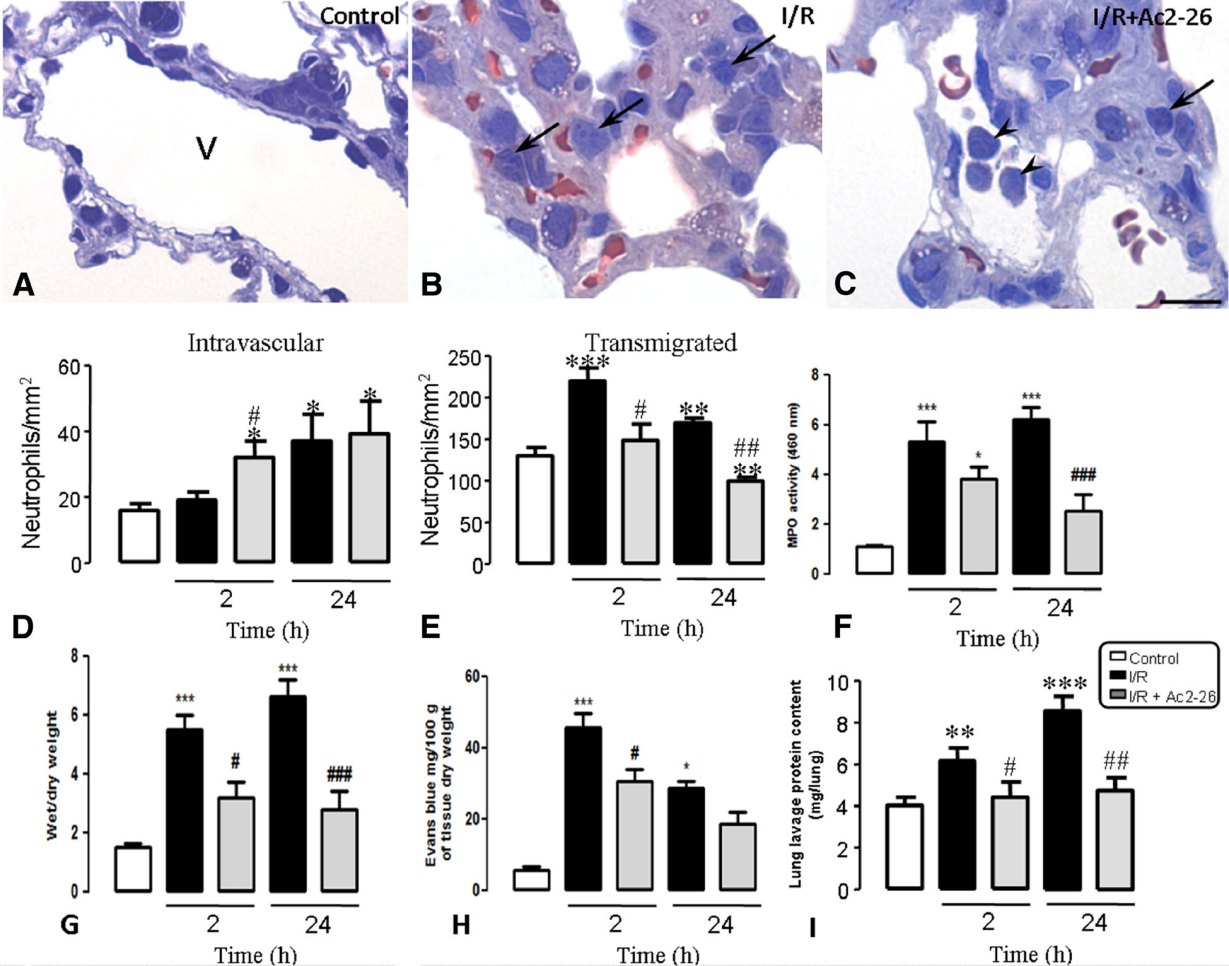
**Histopathological analysis of leukocyte influx into the lung tissue.** (**A**) Histological analysis of the control mice lungs indicated the absence of leukocytes in the vessels (v) and in the lung tissue. (**B**) After intestinal ischemia-reperfusion, several transmigrated leukocytes were observed in the lung parenchyma connective tissue (arrows). (**C**) AnxA1 peptide Ac2-26 treatment prevented leukocytes influx into the lung tissue (arrowheads). Toluidine blue stain. Bar, 10 μm. (**D**-**E**) Cell counting in the histological sections. (**D**) Semi-quantitative analysis showing increase in the intravascular neutrophils after intestinal I/R and after peptide Ac2-26 treatment followed by intestinal I/R. (**E**) Semi-quantitative analysis showing increase in the transmigrated neutrophils into lung connective tissue after intestinal I/R and reduction after peptide Ac2-26 treatment followed by intestinal I/R. Tissue area was determined by Axiovision Software. (**F**) MPO activity analysis demonstrated increased number of neutrophils into lung tissue after intestinal I/R and reduction after peptide Ac2-26 treatment followed by intestinal I/R. (**G-I**) Mice subjected to intestinal ischemia/reperfusion (I/R) had significantly increased pulmonary injury, measured by edema and microvascular leak (hole lung and alveoli). The changes were significantly attenuated by pretreatment with Ac2-26. All data are mean ± SEM from 5 mice per time point. *P < 0.05, **P < 0.01 and ***P < 0.001 versus control group values; #P < 0.05 and ##P < 0.01 versus corresponding intestinal I/R group values.

As depicted in Figure 2 (A-C), neutrophil trafficking from the blood into the lung tissue was affected by intestinal I/R and Ac-2-26 treatment. Figure 2 (panels D and E) shows that neutrophils were present in the lung vessels and connective tissue of the control group (naive group: 18.1 ± 0.5/135.0 ± 10.0; sham group: 18.5 ± 0.6/138.0 ± 15.0; negative control group saline: 19.5 ± 0.7/139.0 ± 12.0; negative control group Ac2-26: 17.6 ± 0.6/130.0 ± 8.0 neutrophils/mm^2^). After 2 h of reperfusion, the neutrophils number in the vascular vessels was not affected, but a higher number of transmigrated cells were observed. However, 24 h post reperfusion, the number of intravascular neutrophils was increased tremendously.

Pre-treatment with Ac2-26 prevented neutrophil migration into the lung tissue, as indicated by the increased number of these cells in the intravascular compartment 2 h after reperfusion. Besides, transmigration of neutrophils, 2 and 24 h after reperfusion was also reduced by prior treatment of mice with Ac-2-26 in comparison to the non-treated group (Figure 2D and E).

Similar to the histological findings, the intestinal I/R induces a large increase in MPO content in lung homogenates (5.5 ± 0.5 and 6.6 ± 0.6 optical density for MPO, respectively 2 and 24 after intestinal I/R) in comparison with animals from control group (naive group: 1.8 ± 0.5; sham group: 1.5 ± 0.4; negative control group + saline: 1.6 ± 0.6; negative control group + Ac2-26: 1.2 ± 0.4) (Figure 2F). This increase was significantly attenuated by the pretreatment of peptide Ac2-26 (3.2 ± 0.5 and 2.8 ± 0.6, respectively 2 and 24 after intestinal I/R) (Figure 2F).

To analyze the pulmonary injury in response to intestinal I/R, we evaluate the Evans blue extravasation (microvascular leakage) and the wet/dry weight (pulmonary edema) technique. The pulmonary injury was significantly higher (5.3 ± 0.8/45.4 ± 4.3 and 6.2 ± 0.5/28.5 ± 2.1, respectively Evans blue measurement and wet/dry weight at 2 and 24 h after intestinal I/R) in comparison with control group (naive group: 1.2 ± 0.2/5.6 ± 1.0; sham group: 1.0 ± 0.3/5.0 ± 1.2; negative control group + saline: 1.2 ± 0.4/6.2 ± 1.6; negative control group + Ac2-26: 1.0 ± 0.5/4.9 ± 1.0) (Figure 2G-H). The pretreatment of peptide Ac2-26 significantly reduced pulmonary microvascular leakage 2 and 24 h after intestinal I/R (3.8 ± 0.5/30.6 ± 2.5 ± 0.7/3.3 and 18.5 ± 3.0, respectively) (Figure 2G-H).

Intestinal I/R mice also had significantly elevated lung plasma leak into alveolar cavity, detected by protein contents in the bronchoalveolar lavage (6.2 ± 0.6 and 8.4 ± 0.6 mg/lungs, respectively 2 and 24 h after intestinal I/R) compared to control mice (naive group: 3.9 ± 0.2; sham group: 4.0 ± 0.4; negative control group + saline: 4.2 ± 0.3; negative control group + Ac2-26: 4.3 ± 0.5) (Figure 2I). Peptide Ac2-26 treatment significantly attenuated the plasma leak in the lung alveolar cavity at 2 and 24 h post intestinal I/R (4.2 ± 0.4 and 4.7 ± 0.6 mg/lungs, respectively) (Figure 2I).

### Effects of Ac-2-26 treatment on the IL-10 and TNF-α and release after intestinal I/R

**Figure 3:**
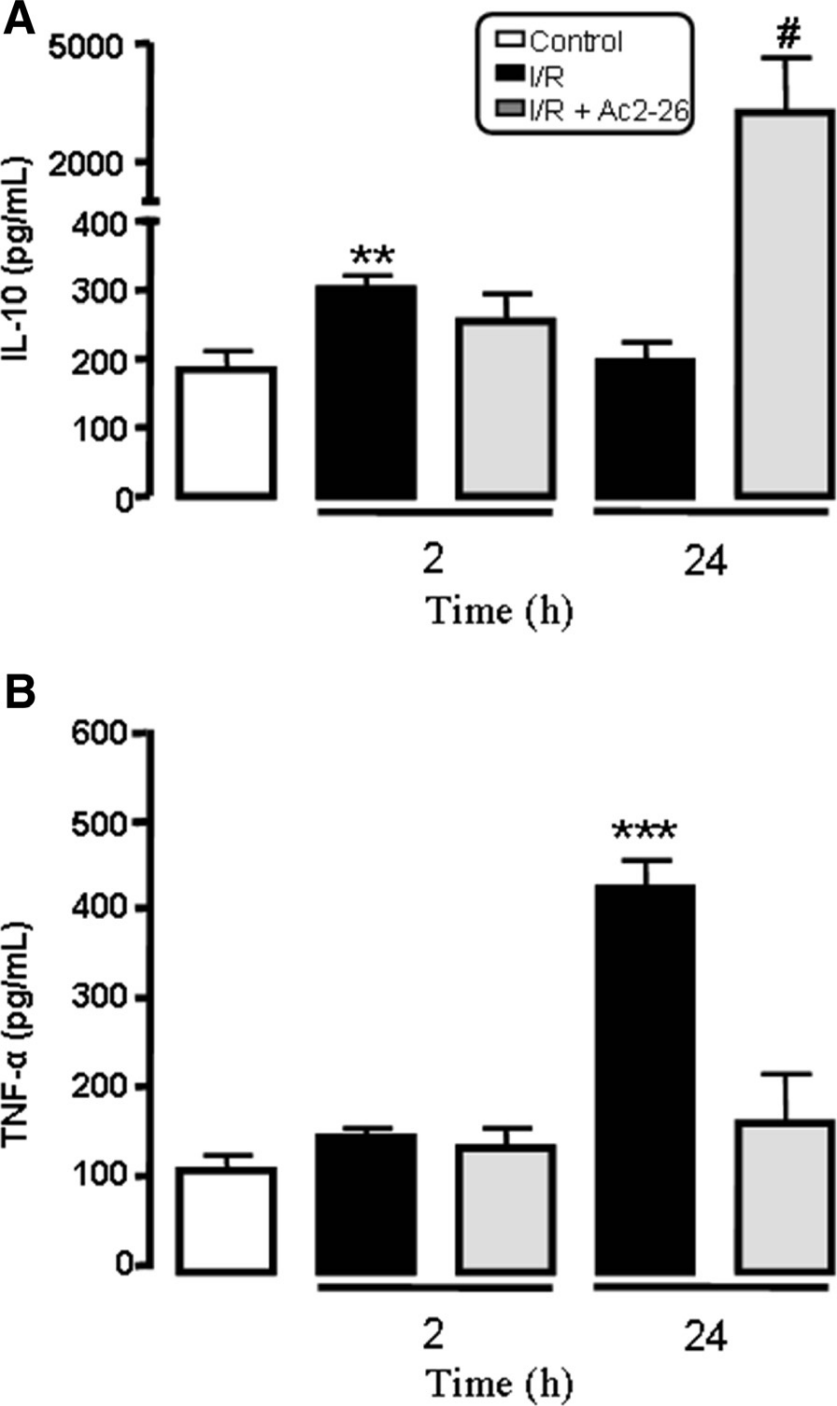
**Plasma IL-10 and TNF-α levels.** Mice (n = 10 per group) were submitted to intestinal ischemia-reperfusion at time 0. The plasma concentration of IL-10 and TNF-α were determined by ELISA. Results are mean ± SEM from two separate experiments with 5 mice per group. **P < 0.01 and ***P < 0.001 versus control group values; #P < 0.05 versus corresponding intestinal I/R group values.

Figure 3 shows increased plasma level of IL-10 when mice were subjected to 2 h of reperfusion, but after 24 h of reperfusion, the level of this cytokine was similar to the control group. The plasma level of TNF-α, a pro-inflammatory cytokine, was elevated after intestinal I/R. Peptide Ac2-26 treatment significantly augmented increase (p < 0.05) in the level of IL-10 while it attenuated increase of TNF-α (p < 0.05) at 24 h of reperfusion when compared to levels found in non treated control mice (Figure 3).

### Effect of intestinal I/R on the annexin-A1 expression in the lung tissue

**Figure 4:**
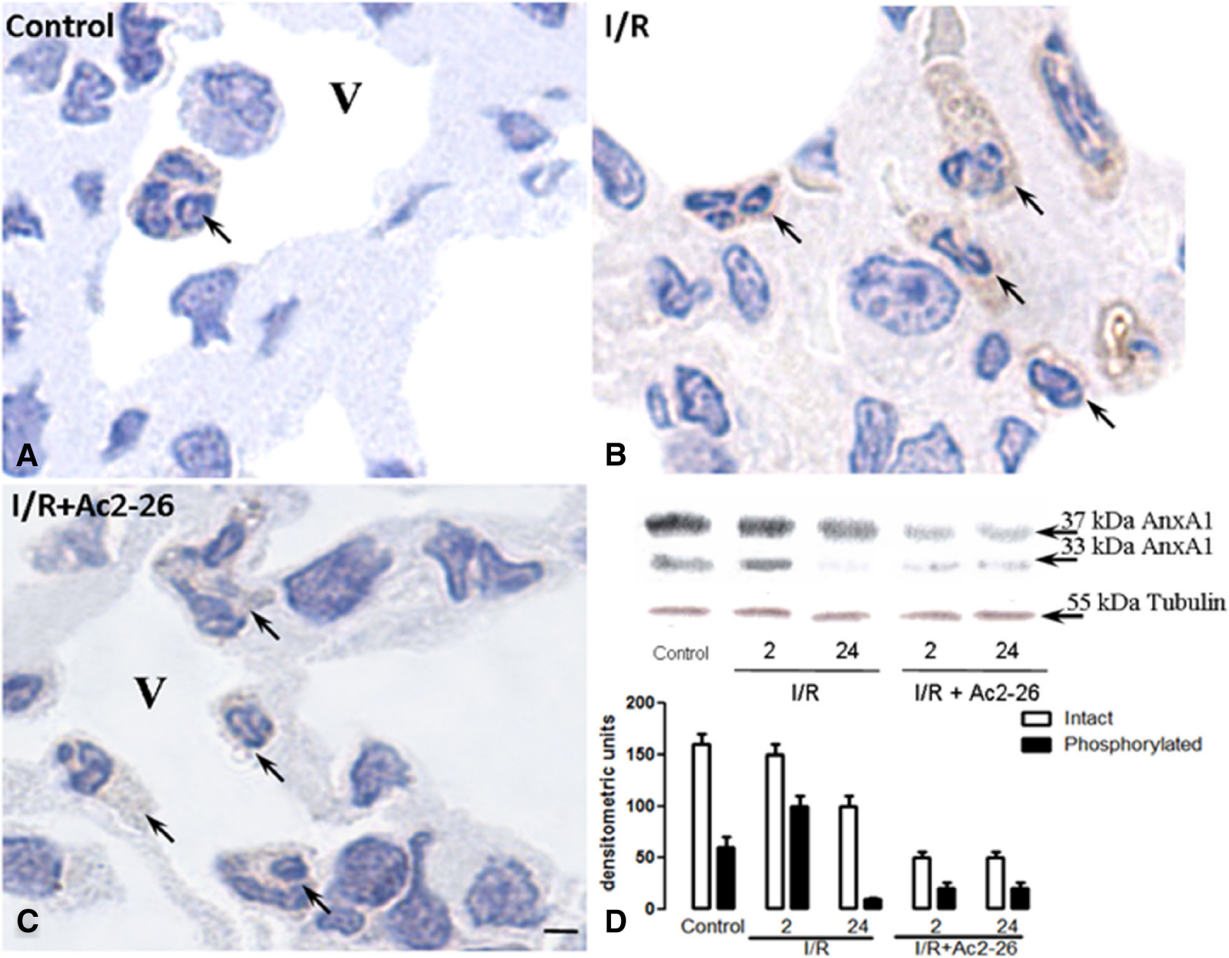
**Endogenous annexin-A1 expression.** (**A**) Leukocytes from control group display basal immunostain for AnxA1 protein (arrow). (**B**) After intestinal I/R, expression of the protein was augmented in the cytosol of lung connective tissue leukocytes (arrows). (**C**) Peptide Ac2-26 pretreatment decreases expression of AnxA1 in the leukocytes (arrows). Vessel (V). Haematoxylin-eosin counterstained. Bar, 10 μm. (**D**) AnxA1 protein content was also analyzed by Western blotting. A representative image of five different experiments indicates that AnxA1 expression (37 kDa; whole protein) in the lung tissue was down regulated after peptide Ac2-26 treatment. The cleaved AnxA1 (33 kDa) was observed in the control groups, increased after 2 h of intestinal ischemia-reperfusion and weakly present after peptide Ac2-26 treatment. Data were analyzed by densitometry as described in material and methods.

Considering the observed pharmacological effects of peptide Ac2-26 treatment, we decided to investigate the expression of endogenous AnxA1 protein in the leukocytes recruited into the lung due to intestinal I/R. The primary antibody used detected both the intact and cleaved AnxA1. However, it was not possible to distinguish between the two by immunohistochemistry. As can be observed (Figure 4), control group of mice displayed basal AnxA1 immunostaining in the cytosol and the membrane of the leukocytes (Figure 4A). After 24 h of intestinal I/R, PBMN, alveolar monocyte/macrophage and migrated neutrophils were highly stained for AnxA1 (Figure 4B and Table 1), but connective tissue monocyte/macrophage and intravascular neutrophils showed a reduction in AnxA1 expression after 2 h of reperfusion (Table 1). After peptide Ac2-26 treatment, a reduction was observed in the AnxA1 endogenous expression in the intravascular (PBMN and PMN) and connective tissue (monocyte/macrophage and neutrophils) leukocytes after 24 h of intestinal I/R (Figure 4C and Table 1).

**Table 1:** AnxA1 Immunohistochemistry analysis in lung leukocytes after intestinal ischemia-reperfusion

	**Control**	**Intestinal I/R**		**Intestinal I/R + Ac2-26**	
		**2 h**	**24 h**	**2 h**	**24 h**
Intravascular PBMN	49,7 ± 7,2	55.9 ± 4.5	83,7 ± 3,1***	65.4 ± 5.3	60,5 ± 5,2#
Alveolar monocyte/macrophage	43,2 ± 5,5	43.1 ± 4.4	66,7 ± 3,7*	44.4 ± 2.7	46,3 ± 7,3
Connective tissue monocyte/macrophage	60,2 ± 1,7	53.8 ± 2.1*	64,9 ± 1,9	45.6 ± 1.8***#	55,8 ±1,9#
Intravascular neutrophils	88,5 ± 4,2	71.3 ± 2.2***	83,6 ± 2,7	72.6 ± 2.5***	65,9 ± 3,0***###
Alveolar neutrophils	----	59.0 ± 3.8	80,6 ± 3,7	47.4 ± 3.4	68,1 ± 3,5
Connective tissue neutrophils	80,9 ± 2,6	71.2 ± 2.3	95,8 ± 2,9***	69.3 ± 2.4*	70,3 ± 1,8*###

AnxA1 protein expression was analyzed in mice lung at 0, 2 and 24 h post-intestinal ischemia-reperfusion (n = 10 per group). Groups of mice were treated with the peptide Ac2-26 (i.p.) as described in Material and Methods section. Values (densitometric units) are mean ± SEM of 10 tissue sections analyzed from five mice per group. *P < 0.05, **P < 0.01, ***P < 0.001 versus control group values. #P < 0.05, ##P < 0.01, ###P < 0.001 versus corresponding intestinal I/R group values.

Finally, Western blotting analyzes indicated increased endogenous AnxA1 expression after intestinal I/R (Figure 4D). However, after the peptide treatment, a reduction in the lung tissue protein content was observed, as the 37 kDa band was less intense (Figure 4D). Interestingly, the AnxA1 cleaved at 33 kDa was highly noticeable at 2 h post reperfusion (Figure 4D), whereas after peptide treatment, this post-translational modification was hardly present in all time-points (Figure 4D).

## Discussion

Intestinal I/R is a risk factor for acute lung injury induction, a lung disease where activated neutrophils play a part [2-4]. In this study, we analyzed the kinetics of leukocyte migration to the lung after intestinal I/R and we assessed AnxA1 expression. We also evaluated the role of Ac2-26, the annexin-1 N-terminal peptidomimetic, on the profile of lung tissue cells and peripheral blood leukocytes.

Our data showed that the period of reperfusion is a crucial factor in the changes to blood cell mobilization. In fact, the early increase of blood neutrophils number 2 h after reperfusion was reverted 24 h later. Thus, it is plausible to suggest that systemic inflammation caused by intestinal I/R caused the increment of blood leukocytes, which migrated to target organ causing acute lung injury. Indeed we [1,15] and others [2-4] have demonstrated an increased influx of neutrophils into the lung and increased microvascular permeability in rodents subjected to intestinal I/R. Here, we observed that intestinal I/R intensified influx of neutrophil into the lung tissue, as evidenced by histological studies. Moreover, previous studies have demonstrated an increased lung myeloperoxidase activity due to intestinal I/R, indicating neutrophils activity at this inflammatory site [15]. These data agree with the concept that intestinal I/R induces remote organ injury, notably in the lung, where endothelial barrier plays a pivotal role in the organ injury [19]. Notwithstanding neutrophils are the principal cells that mediate acute lung injury after intestinal I/R. Mononuclear cells might also contribute to lung changes caused by gut trauma [5], causing immunodepression. Accordingly, innate immune response triggered by intestinal trauma has been associated to induction of lung failure [20]. It is noteworthy to state that our current data revealed a time-dependent decrease of blood monocytes and lymphocytes after intestinal I/R, a fact that was accompanied by their concomitant increase in the lung tissue, indicating, therefore, that these cells were activated by intestinal trauma.

Regarding the involvement of neutrophils in the intestinal I/R-induced remote organ inflammation, some treatments for this condition have been developed. Most of which include neutrophil depletion and direct inhibition of neutrophil activators [5]. However, leukocyte or pro-inflammatory mediator blocking may cause several adverse side effects, because they could also affect activation of the resolution phase of the inflammatory response [14]. Being so, we hypothesize that AnxA1 as a component of endogenous control of inflammatory response could constitute a new approach to control the magnitude of acute lung injury due to intestinal I/R. In fact, our data demonstrated that the AnxA1 mimetic Ac2-26 compound regulated the neutrophil trafficking from the blood vessels into the lung after intestinal I/R, as observed at 24 h time-point. In this scenario, Ac2-26 played a pivotal role in the control of neutrophil influx induced by intestinal I/R as found in other models such as heart ischemia [10,13,21,22]. To test this hypothesis, we assessed the number of intravascular neutrophil and those transmigrated into lung tissue after Ac2-26 treatment upon intestinal I/R. Intravascular neutrophils increased 2 h after Ac2-26 treatment followed by intestinal I/R and remained unaltered 24 h later. Such neutrophil mobilization dynamic was accompanied by a reduced transmigration of neutrophils into lung tissue 2 h and 24 h after reperfusion. Overall, our data suggest that the peptidomimetic Ac2-26 treatment regulates topographic distribution of neutrophils in order to control acute lung injury induced by intestinal I/R. AnxA1 and its peptidomimetic Ac2-26 regulate the leukocyte extravasation/activation through interaction with their receptor, the formyl-peptide receptor (FPR) [13]. Intravital-microscopy studies have demonstrated that AnxA1 does not inhibit the leukocytes recruitment or rolling/adhesion to the post-capillary venules, but affects the cell migration to the inflammatory sites [10,13]. Several studies have demonstrated that the interaction between AnxA1/Ac2-26 and FPR induces a regulation of L-selectin and integrin CD11b expression in neutrophils and monocytes [13,23-25].

AnxA1 action has also been studied in other experimental models of I/R. In the myocardial injury induced by I/R of the left anterior descending coronary artery, the treatment with peptide Ac2-26 or the human recombinant (hr)AnxA1 inhibited leukocyte migration and heart tissue damage [10,13,21,22]. Other models, like renal and cerebral I/R, also demonstrated the protective effect of AnxA1 treatment [26-28]. Moreover, some studies have demonstrated the inhibitory action of AnxA1 in the leukocyte migration to the intestine and cremaster tissue induced by intestinal I/R [28,29].

For a better understanding of other AnxA1 protective effect during the intestinal I/R, we analyzed anti-inflammatory cytokine IL-10 and the pro-inflammatory cytokine TNF-α release. Our data indicated that the peptide Ac2-26 treatment increased IL-10 levels and decrease that of TNF-α in the plasma after 24 h of intestinal I/R. Several works have indicated the induction of IL-10 release after AnxA1 treatment [29-31], and reduction of IL-10 levels in AnxA1 deficient mice [23]. On one hand AnxA1 or peptide Ac2-26 treatment, in the case of LPS-induced endotoxemia, inhibited TNF-α release [31,32], whereas in the absence of AnxA1, increased levels of the cytokine was observed [23]. In addition, antibodies against AnxA1 or an FPR antagonist, BOC-1, caused a reduction in levels of IL-10 in the plasma after intestinal I/R [29]. Also, it is important to highlight that, since endogenous AnxA1 promotes constitutive activation of ERK and innate immune stimulators such as CpG DNA up-regulate IL-10 production in macrophages by activating the extracellular signal-regulated kinase (ERK) pathways, it is conceivable that ERK signaling pathway is involved in the effect of IL-10 up-regulation via AnxA1 administration [33]. All these data indicate the importance of AnxA1 in the induction of the anti-inflammatory cytokine IL-10 release and downregulation of TNF-α level, which highlights the importance of this protein in the regulation of the inflammatory process. All this process might be orchestrated through AnxA1 receptor, FPR/ALX [29].

Finally, we also analyzed endogenous AnxA1 protein expression by immunohistochemistry and Western blotting techniques in the lung tissue after intestinal I/R. Some studies have described endogenous expression of AnxA1 in the airways, in particular alveolar macrophages and lung endothelial cells [23,31]. Our data indicated reduced AnxA1 expression after peptide Ac2-26 treatment. In contrast to our results, previous studies have shown increase in the endogenous AnxA1 after peptide Ac2-26 treatment in leukocytes [13]. This discrepancy can be explained by the high levels of cleaved AnxA1 (33 kDa) observed after intestinal I/R by Western blotting technique. One of the possible post-translational modifications described for these proteins is phosphorylation, which leads to protein translocation to the membrane and release during the inflammatory process [34-37]. Future studies will therefore address the secreted levels of AnxA1 after intestinal I/R.

## Conclusion

In conclusion, our data indicated that AnxA1 peptidomimetic Ac2-26 treatment has both regulatory and protective effect during lung inflammation induced by intestinal I/R. The main mechanisms observed here were the reduction of leukocyte migration into the lung and the induction of the anti-inflammatory cytokine IL-10 release in the blood. The anti-inflammatory effects of AnxA1 as reported herein suggests that the protein could be relevant to understanding mechanisms underlying the pharmacological interventions to control inflammatory events related to intestinal I/R.

## Abbreviations

ALI: Acute lung injury; I/R: Ischemia/reperfusion; ARDS: Adult respiratory distress syndrome; IL-1β: Interleukin-1β; TNF-α: Tumor necrosis factor-α; AnxA1: Annexin A1; BAL: Bronchoalveolar lavage; PBMC: Peripheral blood monocytes; PBS: Phosphate-buffered saline; PBSA: Bovine serum albumin in phosphate-buffered saline; PMN: Polymorphonuclear; IL-10: Interleukin-10.

## Competing interests

The authors declare that they have no competing interests.

## Authors’ contributions

BCG: carried out the experiments. MZ: carried out the experiments. WTL: conceived the study, and participated in its design, coordination and helped in writing of the manuscript. SMO: participated in the study design and participated in sequence alignment and in writing of the manuscript. ASD: participated in the study design, carried out the experiments and participated in sequence alignment and in writing of the manuscript. All authors read and approved the final manuscript.
